# Insight into the emerging and common experimental in-vivo models of Alzheimer’s disease

**DOI:** 10.1186/s42826-023-00184-1

**Published:** 2023-12-11

**Authors:** Rishika Dhapola, Sneha Kumari, Prajjwal Sharma, Dibbanti HariKrishnaReddy

**Affiliations:** Department of Pharmacology, School of Health Sciences, Central University of Punjab, Ghudda, Bathinda, Punjab 151401 India

**Keywords:** Alzheimer’s disease, Animal models, STZ model, Aβ model, APP/PS1, 5 × FAD, Transgenic models, oDGal, APP knock-in

## Abstract

Alzheimer’s disease (AD) is a multifactorial, rapidly progressing neurodegenerative disorder. As the exact cause of the disease is still unclear, the drug development is very challenging. This review encompasses the commonly used AD models involving various chemicals, heavy metals and endogenous substances induced models and the transgenic models. It also provides insight into the reliable emerging models of AD that may overcome the shortcomings associated with available models. Chemicals like streptozotocin, scopolamine, colchicine and okadaic acid render the animal susceptible to neuroinflammation and oxidative stress induced neurodegeneration along with amyloid-β deposition and tau hyperphosphorylation. Similarly, endogenous substances like acrolein and amyloid-β 1–42 are efficient in inducing the major pathologies of AD. Heavy metals like aluminum and fluoride and mixture of these have been reported to induce neurotoxicity therefore are used as animal models for AD. Transgenic models developed as a result of knock-in or knock-out of certain genes associated with AD including PDAPP, APP23, Tg2576, APP/PS1, 3 × Tg and 5 × FAD have also been incorporated in this study. Further, emerging and advanced pathomimetic models of AD are provided particular interest here which will add on to the current knowledge of animal models and may aid in the drug development process and deepen our understanding related to AD pathogenesis. These newly discovered models include oAβ25-35 model, transgenic model expressing 82-kDa ChAT, oDGal mouse and APP knock-in rat. This study may aid in the selection of suitable model for development of novel potent therapeutics and for exploring detailed pathogenic mechanism of AD.

**Graphical abstract:**

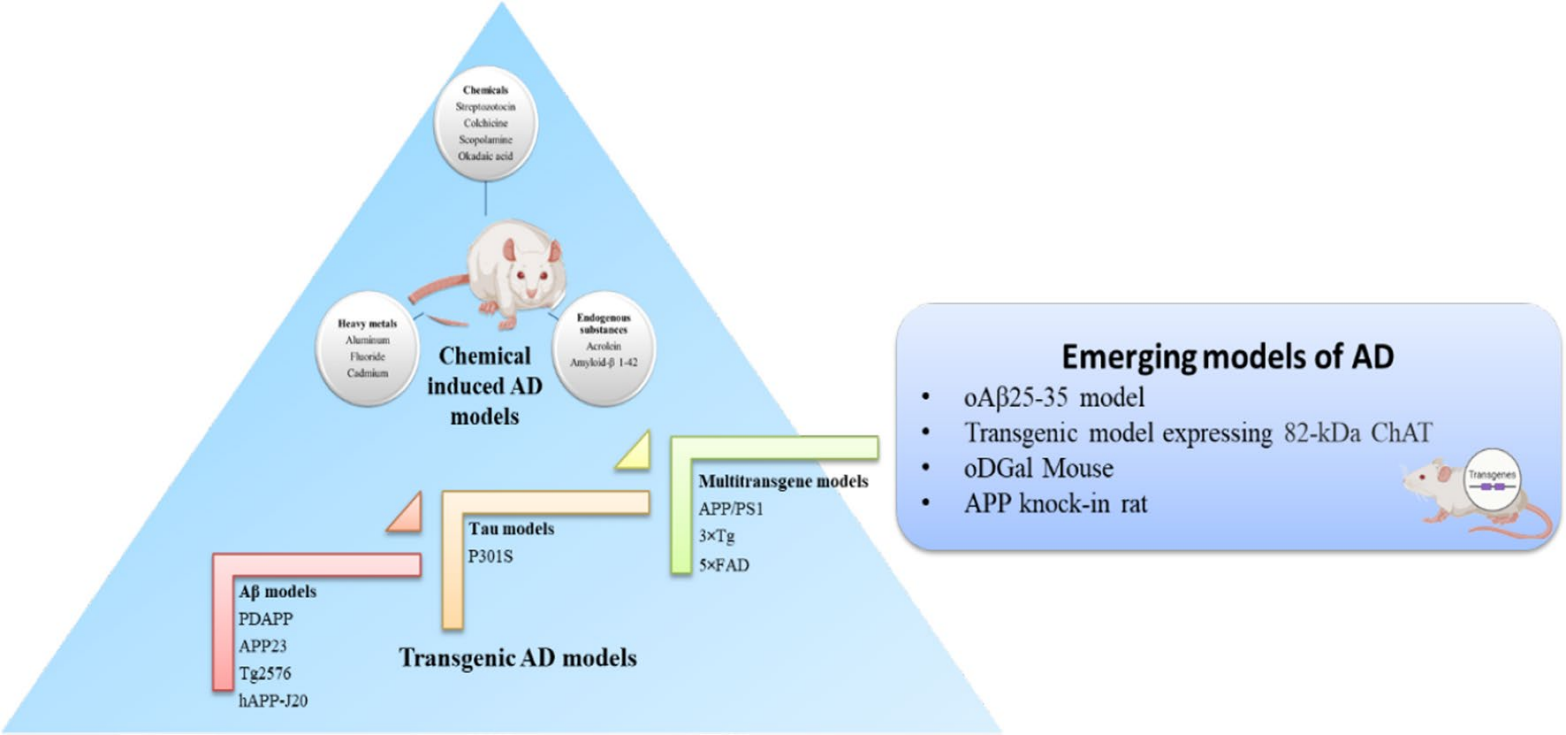

## Background

**Fig. 1:**
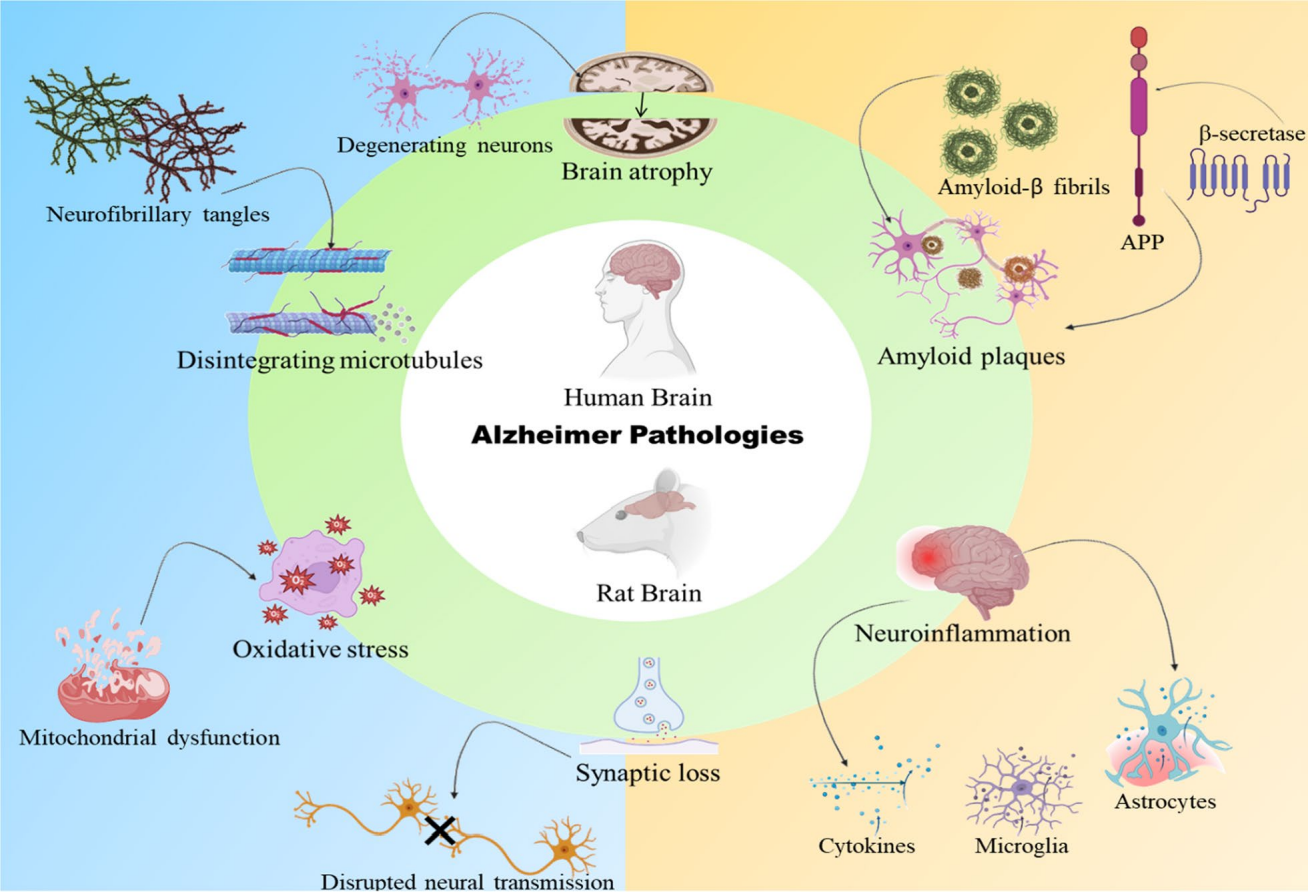
Pathological similarities between human and rodent brain in AD. This figure shows the similarities in the pathologies of rodent brain and human brain during the progression of Alzheimer’s disease. There occurs accumulation of amyloid plaques and formation of tangles along with other hallmarks like neuroinflammation, oxidative stress and synaptic dysfunction. These similarities make the animals suitable for AD research which may aid in the development of potent therapeutics for AD

Animal models play a crucial role in the development of therapeutics and determining the efficacy of a new drug candidate. Alzheimer’s disease is a rapidly progressing neurodegenerative disease which is one of the major cause of dementia around the world accounting for 50–70% of the cases [1, 2]. Animal models are excellent tools for investigating the pathogenic underpinnings of the disease processes as well as for developing novel therapeutic approaches. All existing AD animal models have drawbacks that must be carefully examined before employing them in any study. None of the existing AD models wholly resemble the pathophysiology of AD therefore, the majority of research is conducted to develop models that actively manipulate animals to mimic the disease's symptoms completely. Different aspects of AD have been modeled using a variety of animal species. Rats were initially the preferred species, but over the past ten years, the growing understanding of sophisticated genetic procedures created in mice have encouraged the usage of transgenic models as well [3]. Similarities in the pathology of AD in rodent brain with those in human AD brain make the animals more suitable for AD research (Fig. 1). The major pathologies associated with the progression of AD and other neurodegenerative diseases like amyotrophic lateral sclerosis [4] are Aβ and tau accumulation, neuroinflammation [5, 6] mitochondrial dysfunction [7], oxidative stress [8], ER stress [9], apoptosis [10] and platelet aggregation [11, 12]. Most of the AD cases are early onset or sporadic type whereas the late onset type accounts for only 10% of the cases. Therefore, most of the models rely on the pathogenesis of sporadic AD. Although both the AD types are having similar pathologies including deposition of amyloid plaques, formation of neurofibrillary tangles and loss of cholinergic neurons, the difference lies in the genetic pattern. In familial AD there occurs mutation in the PS1 gene which promotes the formation of diffuse amyloid plaques resulting in the stimulation of Aβ_42/43_ deposition [13]. Various substances are there which are being used as a model to develop AD in the animals. These substances comprise different chemicals, endogenous substances and heavy metals which are toxic when exposed in quantities beyond their permissible range. Compounds that act via recognized disease-modifying pathways enter the clinical studies but somehow fail. This may be due to the compromised data associated with preclinical studies of the compound regarding its target specificity, optimization, and translational properties may be due to improper selection of the animal model. This study describes the common animal models used in Alzheimer’s research and the emerging models which may aid in the drug development process [14].

## Main text

### Animal models of AD

**Fig. 2:**
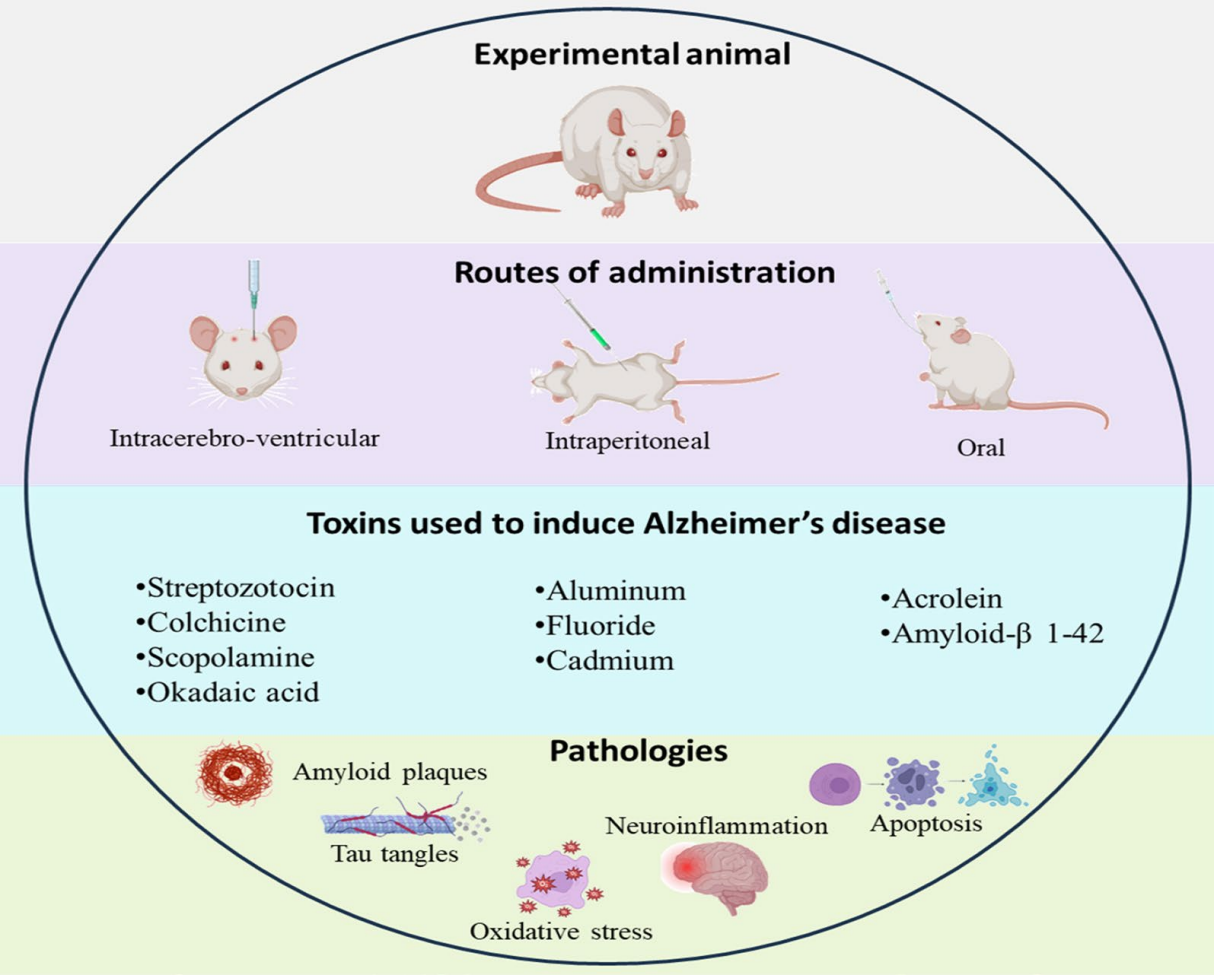
Animals models and their characteristic pathological features. This figure represents various animal models described in this study along with the consequent pathologies induced by different substances. These substances are administered by various routes including intracerebroventricular, intrahippocampal, intraperitoneal and oral. These pathologies subsequently lead to the progression of Alzheimer’s disease

Various substances have been used to induce AD in animals to investigate the efficacy of novel therapeutics and to find out plausible mechanisms involved in the progression of the disease. Based upon the molecular pathways to be studied in the animals the models are selected. Various chemicals, endogenous substances and heavy metals are used for induction of AD in animals which simulate various pathologies of the disease including amyloid plaque deposition, tau hyperphosphorylation, oxidative stress, neuroinflammation, apoptosis and autophagic dysfunction. The mechanism of these substances which leads to AD progression in the animal models, salient features and the timeline are described in Tables 1, 2 and 3 and illustrated in (Fig. 2).

**Table 1:** Characteristics features of various animal models of AD

S. No.	Animal model	Major pathology	Merits	Demerits	Method of administration /Dose	References
1.	Streptozotocin	Neuroinflammation Oxidative stress Biochemical modulations	Induces sporadic AD that is highly prevalent	Long term development of amyloid and tau pathologies No effect on contextual fear memory, High mortality	ICV/ 3 mg/kg	[17, 28, 130, 131]
2.	Scopolamine	Cholinergic dysfunction	Different parameters can be evaluated therefore aids in developing multitarget therapy No Involvement of any surgical procedure	Do not completely mimic AD pathologies Mainly used for preventive AD treatments	ICV/ 2 mg/kg	[28, 32, 36, 132, 133]
3.	Colchicine	Tau hyperphosphorylation	Mimics sporadic AD pathologies Excitotoxicity can also be explored	High mortality Adverse effects	ICV/15 μg/5 μl Orally/0.3 mg/kg	[28, 42, 61]
4.	Okadaic acid	Tau hyperphosphorylation	Similar characteristic pathologies of AD Rapid disease induction	Side effects due to acting upon PP2A that is expressed throughout the body	ICV/70 ng/day	[48, 52, 53, 61]
5.	Amyloid-β1-42	Amyloid-β aggregation Neuroinflammation	Exhibit predictive, face, and construct validity	Neurofibrillary tangles are not seen Adult rodents are used instead of old ones	ICV/ 80 μmol/L Intrahippocampal/ 1 µg/µL	[54, 61]
6.	Acrolein	Oxidative stress Neuroinflammation	Simulates multiple AD pathologies	Typical Aβ plaques as seen in AD individuals are not observed	Intragastric/ 2.5 mg/kg/day	[64, 66]
7.	Heavy metals	Oxidative stress, Neurofibrillary tangles	Ease of aluminum administration Less mortality rates	Plaques pathology is different from AD in humans	Intraperitoneally/ 100 mg/kg Orally/ 150–300 mg/kg	[61, 134]

**Table 2:** Salient features of transgenic animal models of AD

S. No	Model	Transgene	Transgenic promoter	Merits	Demerits	References
1.	PDAPP	APP	PDGF	High pathological similarity with AD patients	Difficulty in standardization and differentiating between functional and pathogenic Aβ	[80, 82, 83]
2.	APP23	APP751 cDNA	Neuron-specific murine *Thy-1*	Hippocampus and neocortex regions are majorly affected as observed in humans	Neurofibrillary tangles are not observed	[84, 85]
3.	Tg2576	APP	Hamster prion protein (PrP)	Slow rate of Aβ deposition	Scant Aβ pathology and plaque burden	[86, 92, 93]
4.	hAPP-J20 mice	Swedish (K670N and M671L); (V7171F)	PDGF	High propensity for thigmotactic swimming thus better to evaluate spatial memory	Neuroinflammation and neuronal cell loss occur before Aβ pathology making it difficult to study plaque development	[95, 96, 99]
5.	P301S	PS19	Murine *Thy1*	Atrophy and damage of hippocampal region makes it clinically relevant to AD patients	No amyloid plaques No link between genetic mutation and tau pathology is found in AD patients	[66, 100, 101]
6.	APP/PS1	APPswe, PS1dE9	Mouse prion protein	Amyloid plaque morphology is similar to humans Homozygous lines are produced	Late onset of cognitive dysfunction No signs of motor deficits	[105, 106, 135]
7.	3 × Tg or LaFerla mouse	APP, PSEN1, MAPT tau	Mouse *Thy1* minigene	Both amyloid plaques and tau tangles can be seen	Evaluation is challenging due to multiple gene stimulation	[107, 110, 112]
8.	5 × FAD	Swedish (K670N, M671L), Florida (I716V), and London (V7171) and human *PSEN1* (M146L and L286V) regulated by *Thy1* promoter	*Thy1* promoter	Prominent amyloid plaque deposition similar to AD patients	No tau pathology is observed	[113, 114]

**Table 3:** Timeline for the emergence of pathophysiological and behavioral hallmarks in various animal models of AD

S.No.	Animal model	Timeline for Pathophysiological alterations	Timeline for behavioral alterations	References
1.	Streptozotocin	21 days	15 days	[20, 21, 136, 137]
2.	Scopolamine	13 days	9 days	[35, 36]
3.	Colchicine	2 weeks	21 days	[138, 139]
4.	Okadaic acid	13 days	10 days	[140, 141]
5.	Amyloid-β1-42	15 days	14 days	[142, 143]
6.	Acrolein	12 weeks	4 weeks	[144, 145]
7.	Heavy metals	25 days	21 days	[146, 147]
8.	PDAPP	6–9 months	3–4 months	[148, 149]
9.	APP23	6 months	3 months	[150, 151]
10.	Tg2576	3 months	9–10 months	[88, 148]
11.	hAPP-J20	5–6 months	9–12 months	[96, 97]
12.	P301S mice	6 months	9–12 months	[101]
13.	APP/PS1	2–3 months	120–250 days	[148]
14.	3 × Tg	6–12 months	4 months	[108, 109]
15.	5 × FAD	2 months old	4–5 months	[152]

### Chemical induced animal models of AD

Chemicals obtained from various sources have been employed since long time for the induction of AD in animals. Based upon the dose, duration and route of administration these chemicals exhibit neurotoxic effect and increase their scope for use in disease induction.

#### Streptozotocin

Streptozotocin (STZ) is a glucosamine-nitrosourea compound acquired from soil bacteria *Streptomycetes achromogenes*. It was originally approved for its anticancer activity. Later studies reported its diabetes inducing ability in experimental models but the dose was around 40 mg/kg of body weight via intraperitoneal route that is way higher than the dose required to induce AD i.e., 3 mg/kg and that too via intracerebroventricular route [15, 16]. STZ have been reported to induce cognitive deficits in animal models [17]. STZ results in aggregation of amyloid beta and increase in GSK3β expression involved in the hyperphosphorylation of tau protein. The pathways that can be targeted for studying new drug candidate in STZ induced animal models comprise of neuroinflammation, oxidative stress and biochemical modulations like increase in GSK-3β levels [17]. STZ efficiently alters both short term and long term memory [18]. STZ has also been found to promote brain insulin alterations as it affects the insulin receptors present in the brain. Altered GSK-3β level is associated with both Alzheimer and diabetes induced dementia progression [19]. Studies on various rat and mouse models have proven Alzheimer inducing potential of STZ. ICV injection of 3 mg/kg body weight of STZ for 21 days exacerbated neuroinflammation, synaptic plasticity dysregulation and amyloidogenesis in Swiss albino mice of 20–25 g and 3 × Tg-AD mice progressing to Alzheimer’s disease [20, 21] Oxidative stress and mitochondrial dysfunction was also seen at this dose in Wistar rats along with alteration in intracerebral glucose metabolism when STZ was administered for 14 days [22, 23]. Single ICV injection of STZ unilaterally in dose 3 mg/kg takes 21 days to develop AD pathologies [24, 25]. In another study 3 mg/kg of ICV-STZ when administered in two alternate days induced AD within 14 days however, when administered bilaterally, pathologies were seen in 21 days [26, 27]. The pathologies associated with STZ induced AD models resemble with the human AD brain pathologies in various aspects. These include, oxidative damage, mitochondrial dysfunction and caspase mediated apoptotic death. Further the Aβ deposition location was similar to that in the AD patients [15]. These studies support the reliable AD inducing potential of STZ. But a major drawback of STZ induced AD model is that it requires surgical precision in administration of STZ in the specific brain regions which is a very tedious process [28].

#### Scopolamine

Scopolamine is isolated from *Atropa belladonna* L and is a tropane alkaloid [29, 30]. It has been used to treat gouty arthritis [31]. Scopolamine is a drug of choice for motion sickness and its derivatives have been used as antispasmodics. Due to its good BBB permeability, it is often used to establish experimental model for neurological disorders. It results in cholinergic dysfunction and amyloid-β accumulation [32]. It is muscarinic receptor antagonist which blocks the muscarinic acetylcholine receptors causing synaptic dysfunction and cognitive impairment [33]. Intraperitoneal injection of scopolamine in rats in a dose of 2 mg/kg/day for 6 weeks increased the levels of accumulated amyloid-β and increased the phosphorylation of tau protein. It exacerbates the expression of GSK3-β affecting the hyperphosphorylated tau levels [34]. Another study using 1 mg/kg scopolamine intraperitoneally for 9 days reported cholinergic insufficiency and oxidative stress due to decreased levels of anti-oxidants like CAT and SOD in the rats [35]. Suggesting the long-term administration of scopolamine induces amyloid plaques deposition and hyperphosphorylation of tau but administration for short duration only stimulates oxidative stress condition and cholinergic dysfunction. Scopolamine when given in a dose of 0.7 mg/kg by IP route increases the expression of AchE and oxidative stress [36]. Reports suggested that this model produces similar disruption of functional connectivity in the brain as observed in AD patients [33]. In a dose of 2 mg/kg it disrupts working and spatial memory and learning within 10 days of administration [37]. However, it does not have profound effect on hyperphosphorylation of tau and Aβ aggregation, rendering this a lacuna of this model [28].

#### Colchicine

Colchicine is an alkaloid that is toxic to the neuronal cells and is derived from *Autumn crocus*. For many years it has been used as an anti-inflammatory drug to treat various inflammation related diseases. The mechanism by which it shows anti-inflammatory activity has been well described in the literature. One of the mechanism is inhibition of microtubule polymerization which interferes in the release of inflammatory mediators [38]. For example, colchicine in a rat model at a dose of 0.3 mg/kg body weight when administered orally twice for 24 h showed anti-inflammatory effects [39]. However, when given via intracerebroventricular route in high doses colchicine has been seen to potentiate neuroinflammation. Colchicine disrupts the stabilization of microtubules and increases neurofibrillary tangles formation which causes cytoskeletal damage and hinders axonal transport. This results in death of neuronal cells specially in the olfactory area, subventricular zone, basal forebrain and dentate gyrus ultimately leading to cognitive impairment. Further it leads to enormous production of ROS, developing oxidative stress condition which exacerbates cognitive decline [40]. Colchicine affects the hippocampal and cortex neuronal health associated with working and reference memory [41]. Colchicine has also been reported to bind with tubulin fibers resulting in tau hyperphosphorylation and microtubule disintegration resulting in hippocampal and basal forebrain cholinergic neuronal death [42].

In Wistar rats, colchicine has been reported to cause neuroinflammation and subsequent neurodegeneration by a single ICV injection in a dose of 15 μg/5 μl. It is also associated with increase in oxidative stress and NO production. In addition it alters the activity of BACE-1 increasing the accumulation of Aβ and stimulates the release of inflammatory cytokines [43]. ICV injection of colchicine in the lateral ventricles of rats in a dose of 15 µg/5 µl of artificial CSF caused neuroinflammation and neurodegeneration by augmenting the release of inflammatory mediators consequently increasing the activity of Cox-2 and synthesis of prostaglandins [44]. In another study involving Wistar albino rats, colchicine in a single dose of 7.5 μg in 5 μl artificial CSF was reported to induce cognitive impairments by increasing the inflammatory markers including pro-inflammatory cytokines, TNF-α and Cox-2 suggesting its neurotoxic effect in varying doses [45]. The clinical similarity of this model is that it affects mainly the hippocampal region of the brain and impairs the memory and learning functions [46]. Disadvantage associated with this model is that a large number of animals is required as the mortality is high and it requires time to develop AD pathologies [42].

#### Okadaic acid

Okadaic acid (OKA) is a polyether C38 fatty acid toxin derived from *Hallichondria okadai*, a black sponge. It is a selective blocker of protein phosphatase1 and protein phosphatase2A involved in tau dephosphorylation and is considered very effective for studying neurotoxicity and other regulatory mechanisms. OKA stimulates hyperphosphorylation of tau protein by increasing GSK-3β expression and results in the formation of neurofibrillary tangles progressing AD pathology [47]. It also induces oxidative stress, neuroinflammation, glial activation, cholinergic dysfunction, glutamate excitotoxicity, and mitochondrial dysfunction. 10 nM to 1 μM OKA was used to induce Alzheimer’s disease in Zebra fish which resulted in cognitive decline of the fishes [48]. In rats 200 ng/kg of ICV-OKA interfere with the expression of MAPK1/3 and MAPK14 that are involved in the regulation of tau phosphorylation [49]. In another study, 2 µl OKA dissolved in DMSO was injected in the hippocampus at a concentration of 0.2 µM using artificial cerebrospinal fluid for dilution. OKA induced memory and cognitive dysfunction resulting due to decreased expression of BDNF in the rat hippocampus. The PI3K/GSK-3β/Akt pathway is considered to be involved in this discrepancy related to BDNF [50]. 70 ng/day administration of OKA for 14 days in hippocampal region of the brain unilaterally shoed NFT formation and cognitive decline [51]. Similar to AD patients, the activity of protein phosphatases (PP2A) is reduced in this model which leads to accumulation of hyperphosphorylated tau protein. Along with this it produces oxidative stress, neuroinflammation and neurotoxicity [52]. The disadvantage of this model is that it does not develop amyloid pathology associated with AD which will hinder the evaluation of the effect of novel drug candidate on amyloid plaque build-up [53].

### Endogenous substances induced animal models of AD

There are some endogenous substances which have the potential to induce AD in animals. Commonly used substance is Amyloid-β 1–42 which causes degeneration of neurons in the brain regions responsible for cognitive functions and promotes amyloid plaque deposition. Another recently developed animal model is acrolein induced AD model which results in neurodegeneration and cognitive impairment in the animals. These models have been described herein.

#### Amyloid-β 1–42

It has been shown that the Aβ_1−42_ fibril exhibits significant toxicity when administered in-vivo because it results in greater pathophysiological damage than the Aβ_1−40_ fibril. Aβ_1−42_ peptide is therefore thought to be a powerful stimulator of neuroinflammation and other pathogenic aspects of AD like oxidative stress [54]. Aβ_1–42_ aggregation is the major hallmark for Alzheimer’s disease which further triggers the progression of the disease. Rats are administered with 80 μmol/L of Aβ_1–42_ intracerebroventricularly diluted with 5 µl double distilled water. A total of 8 doses in the study are given every other day at a rate of 1 µl/min for 5 min. Aβ oligomers have been seen to induce synaptic disruption, neuroinflammation which leads to degeneration of neurons and ultimately cognitive decline [55]. When Aβ_1–42_ was administered by intrahippocampal route, it induced neuroinflammation and upregulated APP expression along with decreasing the expression of protein phosphatases. These all pathologies have been observed in AD patients too [8] making this a clinically relevant model of AD. Neuronal loss was also observed in the experimental animals. Using the Stereotaxic co-ordinates: 3.6 mm posterior to the bregma, 2.4 mm left/right to the midline and 2.8 mm ventral to the bregma Aβ_1–42_ was injected on each side of the hippocampus with a volume of 1 μL containing 4 μg Aβ_1–42_ [56]. In another rat model Aβ_1-42_ was given 5 µL in a concentration of 1 µg/µL in sterile saline solution in the lateral ventricles. The co-ordinates used were left, relative to the bregma; 0.8 mm posterior, 1.2 mm lateral [57]. Single i.c.v. injection of Aβ_1−42_ in a dose of 4 µl also induced neurodegeneration in animals [58]. The disadvantage associated with the model is that the sudden induction of the disease does not allow much similarity to the human AD. Further, there is a need of good surgical skills and precise administration is required [59, 60]. Moreover, Neurofibrillary tangles are rarely observed and adult rats are used instead of old ones [54, 61].

#### Acrolein

Acrolein is a neucleophilic α, β‐unsaturated aldehyde. It is found as an endogenous substance in human body [62]. Acrolein is a component of reuterin which is an organic compound and a potent source of acrolein. It is produced by gut microbiota when glycerol is present. It can also be formed by hydroxyl amino acids when these are acted upon by myeloperoxidase in the presence of hydrogen peroxide and chloride ion. Moreover copper dependent amine-oxidation of spermidine and spermine is also a source for acrolein [63]. It has been reported that there is increased level of acrolein in AD brains. In a mouse model it was seen that acrolein administration induced cognitive impairment along with deposition of Aβ and increased phosphorylation of tau. In addition it stimulated microglia and astrocytes resulting in neuroinflammation and synaptic dysfunction [62]. Intragastric administration of acrolein by gavage in a dose of 2.5 mg/kg/day for 8 weeks resulted in fluctuation in the level of oxidative markers like superoxide dismutase and malondialdehyde. Further cortex and hippocampal BACE1 activity was found to be increased along with decrease in the expression of A disintegrin and metalloproteinase domain containing protein 10 (ADAM-10) involved in the proteolytic cleavage of APP which prevents Aβ generation [64]. In another study acrolein in a dose of 3 mg/kg/day for 2 weeks induced oxidative stress in rats leading to neurodegeneration. It decreased the levels of anti-oxidants and activated MAPK pathway Acrolein has also been found to induce tau hyperphosphorylation by activating JNK/p38/ERK1/2 pathway along with increase in Aβ concentration [65]. The disadvantage of this model is that typical Aβ plaques as seen in AD individuals are not observed in acrolein induced AD models [66].

### Heavy metal induced animal models of AD

Environmental risk variables including heavy metals have a significant effect on the progression of AD and associated dementia. Lead, cadmium, and manganese are potent neurotoxic components that result in AD upon prolonged exposure. Although manganese is a vital element required for neuronal survival, it has been reported to exhibit hazardous effect when exposed to excessive amounts. Therefore, due to their neurotoxic effect these heavy metals have been used to induce AD models that have been described herein [67].

#### Aluminum

Aluminum has been recognized as a neurotoxic substance since ages. Its intake has been reported to generate pathological hallmarks that are associated with the progression of AD. Al exposure has been reported to cause cholinergic dysfunction which eventually leads to synaptic dysfunction and cognitive impairment [68]. Aluminum chloride easily enters the brain via BBB and accumulates there mainly in the hippocampus. In a study, rats were orally administered with 50, 150 and 450 mg/kg of aluminum for 90 days and it was observed that the mRNA levels of proinflammatory markers including IL-1β, IL-6, TNF-α and MHC II were increased. Also, the expression of neuronal survival proteins like BDNF was found to be decreased. This resulted in deformed synaptic plasticity and impaired cognitive functions [69]. Another study reported that when rats were intraperitoneally administered with aluminum chloride at a dose of 100 mg/kg bw for 60 days, it stimulated the expression of acetylcholinesterase. APP and gamma secretase activity was also enhanced which increased the levels of accumulated amyloid-β in the hippocampus and cortex region of the brain [68, 70, 71]. In Wistar rats, aluminum chloride when given orally in a dose of 150 mg/kg/day for 90 days exerted neurotoxicity by stimulating neuroinflammation, oxidative stress and amyloid-β accumulation in the hippocampus of the rat brain. The similar pathologies have been observed in AD brains [5]. It also decreased the levels of anti-oxidants like superoxide dismutase [72]. In another rat model AlCl3 in a dose of 300 mg/kg body weight for 10 weeks induced cognitive impairment by causing oxidative stress, cholinergic insufficiency, amyloid plaque deposition and neurofibrillary tangle formation [73]. This suggests that aluminum can be used to induce AD in animal models for studying the safety and efficacy of various developing drugs. The amyloid-β plaques and NFTs have not been observed much in aluminum induced model rendering it a limitation of this model [66].

#### Fluoride

Foods, water, air, additives, industrial effluents, pesticide residues, and some medications are contributors of fluoride ingestion. Sodium fluoride has been found to induce neurotoxicity in the rat offspring when NaF in 3 doses 25, 50 and 100 mg/L in drinking water from the day of pregnancy till 21st day post-delivery was administered. NaF results in apoptosis, disturbs autophagic flux by decreasing the expression of autophagosomes and lysosomal fusion proteins like ATG14 and SNARE resulting in cognitive deficits [74]. In a study when rats were administered with 15 mg/L of sodium fluoride (NaF) in drinking water for 45 days it induced cholinergic deficits and oxidative stress. In addition, acetylcholine levels were found to be decreased in the brain areas involving hippocampus, cerebrum and cerebellum [75]. It has also been reported that when pregnant rats were exposed with 5 and 10 mg/L of fluoride, offspring has motor deficits [76]. Similar to AD patients the antioxidant activity of the AD model brain is compromised leading to oxidative stress and AD progression [77].

#### Mixture of heavy metals

Mixture of heavy metals has been observed to be a potent strategy for inducing AD model. In a study, rats were treated orally with the combination of aluminum, cadmium and fluoride in a dose of 50 mg/kg, 5 mg/kg and 20 mg/kg respectively for 90 days. This led to neurotoxicity in the rats induced by the generation of free radicals and overexpression of inflammatory mediators. The level of neuroinflammatory cytokines has been detected to be increased in post mortem AD brains suggesting the clinical relevance of this model [78]. In addition, heavy metals elevate the deposition of Aβ and tau tangles along with increase in the expression of AChE and monoamine-oxidase (MAO) enzyme [78]. In another rat model, a mixture of aluminum chloride and iron was administered to induce AD like pathologies. AlCl_3_ in a dose of 100 mg/kg and iron in a dose of 120 µg/g were given orally for 28 days which resulted in oxidative stress, cytokine storm, dyshomeostasis of neurotransmitters level and other biochemical modulations. It also affected the levels of accumulated amyloid-β and hyperphosphorylated tau. The expression of NF-κB and caspase-3 were also found to be altered which indicates the stimulation of neuroinflammation in the brain of the rats treated with AlCl_3_ and Fe [79]. The disadvantage of this model is that amyloid plaques pathology is different from that in human AD brains [61].

### Transgenic animal models of AD

**Fig. 3:**
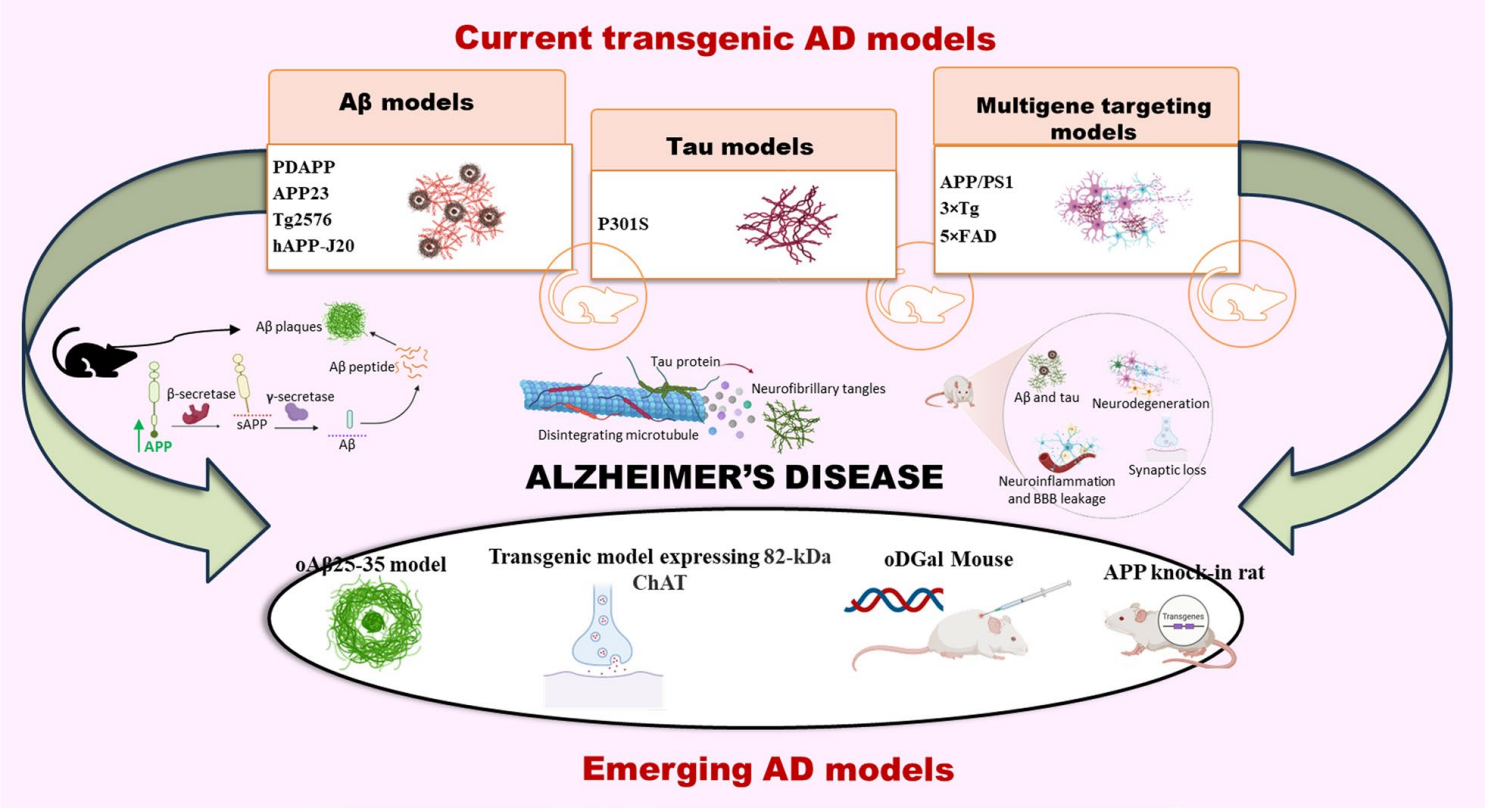
Transgenic and emerging models of AD. There are various knock-in and knock-out models of AD which overexpress certain genes related to AD pathology. Models like PDAPP, APP23, Tg2576 and hAPP-J20 overexpress APP gene to produce increased amount of Aβ along with other pathologies. P301S model exhibits increased tau hyperphosphorylation and models including APP/PS1, 3 × Tg and 5 × FAD show various pathologies altogether comprising Aβ deposition, tau hyperphosphorylation, neuroinflammation and other pathologies. Further figure shows newly discovered models which mimic the AD associated pathologies resulting in the development of reliable model for studying the pathogenesis and therapeutics for AD. These models include oAβ25-35 model, transgenic model expressing 82-kDa ChAT, oDGal mouse and APP knock-in rat

As the major hallmarks of the disease are amyloid-β accumulation and tau hyperphosphorylation, various approaches are made to attain the desired pathological features by genetically developing models possessing the genes promoting the specific pathologies. Salient features of most commonly used transgenic models have been considered herein along with the merits and demerits associated with these models (Fig. 3) (Table 2).

#### PDAPP mice

It was the first transgenic AD model which was related to increased level of Aβ_1-42_ by several folds in the brain. To establish this model FAD mutated transgene containing AD models were employed, in which this transgene was over expressed. Platelet derived growth factor-β (PDGF) was used as promoter for stimulating APP having FAD related mutation (V717F). Unrelated to its name, PDGF is abundant in the brain too. The resulting model acquired 18 times increased APP RNA and 10 times increase in human APP protein that eventually increased the Aβ levels [80].

The pathological hallmark of this model is increase in the level of accumulated Aβ1-42 in the cortex and hippocampus regions of the brain. This Aβ deposition leads to neurite degeneration and activation of glial cells including astrocytes and microglia which furthers initiates other complications like neuroinflammation which goes with the age [81].

The advantages of this model is that the pathological condition arising as a result of Aβ accumulation including tau hyperphosphorylation, synaptic dysfunction and BACE1 over-expression are very similar to human AD pathologies [82]. Further, when developing the treatment for AD, this model will help in establishing the effect of novel therapeutic compound in γ-secretase and BACE-1 activity. Although this model comes up with lots of merits, it possesses demerits too which include the difficulty in distinguishing the activity of functional Aβ and over-produced Aβ and unlike humans, cognitive decline sometimes occurs way before the accumulation of Aβ. Further, standardization of this model is also troublesome as different strains, varying promoters and transgene constructs are employed [83].

#### APP23 mice

The APP23 transgenic mouse model was first reported by Sturchler-Pierrat and colleagues. To establish this model the expression of human APP751 cDNA which incorporated Swedish double mutation (K670N/M671L) was triggered by using neuron-specific murine Thy-1 as a promoter. Originally C57BL/ 6xDBA2 background was used, but then constantly backcrossed to C57BL/6. The resulting model over expresses the transgene for APP seven folds in comparison to the wild type mouse. The increased APP expression was observed predominantly in the hippocampus and neocortex region of the brain involved in AD pathology [84].

APP23 transgenic mice are characterized by the build-up of amyloid plaques which are more rigid and keep on accumulating exponentially with age. It has been reported that Aβ plaque buildup is more prompt in female mice than in male ones. Other complications like neuroinflammation, synaptic dysfunction, neuronal loss and hyperphosphorylated tau has also been observed along with Aβ. Aged APP23 mice were reported to have cholinergic insufficiency and degenerated neurons in the CA1 hippocampal region [85]. The drawback of this model is its inability to develop the major hallmark of the disease i.e., neurofibrillary tangles.

#### Tg2576 mice

The Tg2576 mouse model of AD exhibits double Swedish mutation of APP with hamster prion protein (PrP) promoter. This mutation results in increased synthesis of Aβ42 and Aβ40. Studies revealed that as these mice undergo aging, amyloid plaques similar to those observed in AD patients’ brain are seen. Further, behavioral and cognitive deficits has also been observed in Tg2576 mice which progresses with the age [86].

The pathologies associated with this model include amyloid plaques formation and neuroinflammation [87]. There is deficiency of antioxidants like GPX1, SOD1, and SOD2 in the neocortex region of Tg mice at 3 months of age. PGC1-α, the major regulator of these anti-oxidants, is also less expressed in the neocortex of Tg2576. Further, pathologies associated with the mutation are more prominent in female Tg2576 mice than in male mice [88]. In addition to these, gliosis and astrocytosis has also been reported as the mice reach 12 months of age. Long-time retention of amyloid plaques in the cerebral cortex region of the brain disturbs glucose metabolism which leads to neurodegeneration [89].

The Tg2576 model has a substantial benefit that neuritic plaques start to develop at about 6–7 months and increases exponentially with age. Additionally, these age-related alterations develop at the same time as cognitive deficiencies, indicating that these mice can be used to study Aβ-modifying therapeutics ameliorating cognitive deficits [90]. Another advantage is that these mice exhibit clinical traits that are similar to those of AD patients facilitating researchers to study the disease and the mechanisms involved more precisely [91]. Relatively slow rate of Aβ deposition is considered to be both the merit and demerit of this model [92]. in some studies, even after 22 months Tg2576 mice reflected only scant Aβ pathology and plaque burden [93]. Therefore the major disadvantage is that the AD phenotype is developed late in Tg2576 models [94].

#### hAPP-J20 mice

The hAPP-J20 mouse model of AD expresses Swedish (K670N and M671L) and Indiana (V7171F) mutations on a C57Bl/6 × DBA2J background with PDGF as promoter [95]. As these mice attain the age of 5 months, they begin to develop amyloid plaques due to increased expression of Aβ1-42 which aids in studying pathologies associated with Aβ. This AD model is characterized by recognition and spatial memory deficits in-line with amyloid plaque deposition in the hippocampus and cortex region of the brain [96].

By the age of 5–6 months, J20 mice develop Aβ plaques in the hippocampus and exhibit long-term memory deficits [97]. Over-activation of astrocytes and reactive microglia have been seen in these models that results in neuroinflammation [96]. Trans-arachidonic acid (TAA) may be employed as an oxidative stress biomarker of AD since the production of endogenous TAA may be ascribed to Aβ-induced nitro-oxidative stress in AD brain. Supporting this a study revealed that the level of endogenous TAA in the hippocampus of J20 mice was significantly higher than the other mice used in the study i.e., B6 mice. A positive correlation was found between TAA level and the disease progression [98].

The advantage of this model is that it can be used for better evaluation of spatial memory with the help of Morris Water Maze since it has high propensity for thigmotactic swimming [99]. As far as the disadvantage is concerned, tau hyperphosphorylation has not been seen in these models. It has been noted that neuronal cell loss and inflammatory response occur way earlier than the deposition of amyloid plaques in these models that makes it difficult to study the development of amyloid pathology [99].

#### P301S mice

P301S mice exhibiting tauopathy contains P301S mutation expressing the 383 aa isoforms of human tau regulated by murine thy1 promoter. This model exhibits increased tendency to form tau tangles or neurofibrillary tangles. With age these mice out-show other pathologies including motor abnormalities and paraparesis [100]. When the mice attain 6 months of age, tau tangles have been seen aggregated in the brain along with atrophy of hippocampus and entorhinal cortices as the mice reaches 9–12 months of age. Further, synaptic loss has been reported in the hippocampus region in 3 months of age followed by microglial activation resulting in neuroinflammation [101]. The disadvantage of this model is that amyloid plaque formation is not observed in the brain. Further, no association is seen in AD patients between the genetic mutation and tau pathology [66].

#### APP/PS1

A human/mouse chimeric amyloid precursor protein and a human presenilin-1 are expressed together in APP/PS1mice, and both of these proteins possess mutations linked to familial AD. These mutations cause persistent amyloid-β accumulation, neuroinflammation, and cognitive decline. Only single mutation of APPswe was not efficient for generating Aβ deposits. It was seen that it takes around 24 months to develop visible amyloid-β aggregates when there was only APPswe mutation. However, co-expression of PS1dE9 augmented Aβ42 deposits within 6 months of mice age [102].

This model shows neuroinflammation associated with amyloid plaques along with synaptic dysfunction. Aβ is believed to be one of the main factors contributing to the prolonged inflammatory reaction in the AD brain, although the specific molecular pathway by which Aβ exhibits its deteriorating effect is yet unknown. In the region of senile plaques, Aβ buildup and agglomeration can trigger the onset of inflammatory responses and stimulate microglia and reactive astrocytes [103].

When compared to other AD mouse models APP/PS1 mice exhibits higher number of plaques and substantially larger [104]. The possible advantages of this model are: amyloid plaques can be seen earlier and the build-up is also rapid; the morphology of the formed plaques resembles to that in humans; variability internally among the animals is not seen and there is no difference in the pathologies between both the genders; and, high chances of production of homozygous line [105]. However, this model also comes with some drawbacks including late onset of cognitive dysfunctions which occurs at around 11 months of age. In addition, there is no sign of motor deficits associated with AD in APP/PS1 model [106].

#### *3* × *Tg or LaFerla mouse*

The 3xTg-AD mouse was developed in 2003 possessing three familial AD mutations: the Swedish *APP* mutation, the *PSEN1* M146V mutation, and the *MAPT* P301L mutation regulated by a mouse *Thy1* minigene whereas expression of mouse *Psen1* with the M146V mutation is regulated by the cognate mouse [107].

The 3xTg-AD mouse builds amyloid plaques and neurofibrillary tangles in the similar pattern as observed in humans which may be useful for examining the pathophysiology involved in AD. When mice attain 6 months of age the long-term potentiation is affected, however, cognitive dysfunction begins at the age of 4 months [108]. Some studies revealed that the build-up of Aβ plaques was observed at 6 months of age and neurofibrillary tangles were formed within 12 months. But it was seen that synaptic loss occurs way before these pathologies. However, recent studies demonstrate that at 12 months of age, male mice have low to no plaque and tangles, whereas female mice retain plaques at 6 months and NFTs at 12 months of age [109].

Advantage of this model is that both the pathologies plaque deposition and tangle formation can be seen together [110]. Furthermore, the onset of these pathologies is way similar to that observed in human AD patients. Similar to humans the plaque deposition is initially detected in the hippocampus and amygdala region of the brain followed by tau tangles that occur at 12 months of age in the limbic areas [111]. However, mutations in the Mapt gene encoding for tau protein is associated with parkinsonism instead of AD. Additionally, the data are challenging to evaluate due to the anomalies caused by the stimulation of numerous genes [112].

#### *5* × *FAD*

The 5XFAD transgenic mouse was developed in 2006. This mouse possesses three FAD mutations [the Swedish (K670N, M671L), Florida (I716V), and London (V7171) mutations] and human *PSEN1* with two FAD mutations (M146L and L286V) regulated by *Thy1* promoter [113].

The major pathologies associated with 5XFAD mice are amyloid plaque build-up, gliosis, and neuronal loss along with cognitive and motor deficits [114]. Intraneuronal Aβ-42 builds up in puncta that co-label for Transferrin receptor and LAMP-1 in the soma of 5XFAD neurons which signifies the localization is in endosomes and lysosomes, respectively. Further, caspase-3 activation is also seen in these models in the soma and dendrites of Aβ42 marked neurons signifying neuronal death potentiated by apoptosis [115].

Amyloid pathology, the major hallmark of AD, is promptly reconstructed by 5XFAD mice, which may serve as effective models for intraneuronal Aβ-42-induced neurodegeneration and amyloid plaque development [114]. The major disadvantage of this model is that it doesn’t reflect tau pathologies which makes it significantly different from human AD pathology [113]. Tau pathology gradually results in neurodegeneration and cognitive deterioration unlike amyloid plaques which promptly cause neuronal death [116].

To overcome this a new model, 6xTg, has been developed in which the expression of the transgene (Tau) was elevated bit higher than the parental line. This model shows both the AD associated pathologies including Aβ plaques build-up (within 2 months) and tau deposition (within 4 months) in a single model. Further, Aβ accumulation and NFT formation were more in cortex than in the hippocampus [116].

### Emerging animal models of AD

There is a need to develop new models for studying the pathogenesis of AD as the current models by some or other ways lack behind in establishing a perfect AD model. Therefore, here are some newly discovered models that can be studied further to develop a reliable model for the pathological and therapeutic studies of AD (Fig. 3).

#### oAβ25-35 model

Aβ25-35 is the smallest fragment of amyloid-β that acquires a β-sheet configuration resulting in aggregation. This fragment shows comparable neurotoxicity to Aβ40 and Aβ42 [117]. Therefore, this is now being considered to develop new model for AD. In-vivo studies have revealed that the pathologies including cognitive deficits, synaptic dysfunction, neurite atrophy and neuronal death are quite similar to that of Aβ40 and Aβ42 [118]. Therefore, it can be conferred that injection of oligomeric Aβ25-35 (oAβ25-35) could initiate a sequence of harmful events, which includes the induction of endogenous Aβ production and tau hyperphosphorylation resulting in AD-like pathology. This model demonstrates characteristics that are strikingly similar to AD including neuroinflammation, oxidative stress, excitotoxicity, synaptic loss and cognitive decline. The acute oAβ25-35 model appears to be particularly relevant for understanding the mechanisms taking place during the early stage of AD, which starts decades before the onset of the first clinical symptoms [119].

#### Transgenic model expressing 82-kDa ChAT

There is a vast involvement of cholinergic deficiency in the pathogenesis of AD in which there occurs fluctuation in the activities of enzymes involved in acetylcholine production and degradation. It has been seen that there is increase in the activity of acetylcholinesterase (AChE) and butyrylcholinesterase (BChE) and decrease in the activity of choline-acetyltransferase (ChAT) [120]. Aging, moderate cognitive impairment (MCI), and AD involves the disrupted regulation of the nuclear distribution of 82-kDa ChAT, which shifts to cytoplasmic localization [121]. The selective sensitivity of cholinergic neurons, which are well known to be especially vulnerable to the deterioration seen in AD, may be attributed to 82-kDa ChAT as a result of these temporal/spatial alterations [122]. Considering this a successful transgenic mouse model has been developed that expresses neuronal 82-kDa ChAT by employing Cre-lox recombination system and Nkx2.1-Cre driver mice. The basal forebrain neurons mostly produced the 82-kDa ChAT mRNA and protein, and its intracellular localization replicated the age-associated trend originally observed in human necropsy brains. The cognitive and inflammatory characteristics of older 82-kDa ChAT-expressing animals were improved. However, its role in age-related microglial function needs to be thoroughly studied. Therefore, this transgenic mice can be used as a novel model to study cholinergic dysfunction associated with AD [121].

#### oDGal mouse

Sporadic AD (sAD) is a dementia-causing condition that develops gradually. The disease's symptoms start off slowly and get severe as time passes. A global economic load, sAD is defined by a steady decline in cognitive abilities that results from numerous diseases. The complex interactions between multiple genetic, epigenetic, proteostatic, and environmental variables lead to the etiology of sAD [123]. Currently, the available transgenic models focus on mimicking familial AD symptoms which cannot exhibit sporadic AD symptoms as they both differ symptomatically. Therefore, it’s the need of the hour to develop a reliable model for studying sporadic AD pathologies and establish its treatment. Therefore, to overcome these shortcomings, a new murine model, oligomeric DGal (oDGal), has been developed that outline common AD pathologies along with behavioral alterations. D-galactose is a nutrient required for the generation of AGE (advanced glycation end-products). It is hypothesized that AGE buildup, a prevalent sign of aging that is exacerbated in many peripheral and neurological illnesses, will hasten oxidative damage [124]. Administration of Chronic D-galactose by i.p. route has already shown increased natural aging in different mouse strains. It leads to learning and memory deficits along with decrease in acetylcholine levels [125]. In a study chronic oGAL has been administered via drinking water employing C57Bl6/j mice. Following antioxidant therapy, cognitive decline and the appearance of pathology were delayed, which indicates ROS as the molecular trigger of subsequent pathologies making this model a reliable model for sporadic AD. The pathologies associated with this model were found to increase AGE levels resulting in increased ROS, hyperphosphorylated tau and cognitive decline. Further, due to elevated oxidative stress, BACE1 activity is also stimulated. This approach is useful because it enables the analysis of both prodromal and symptomatic sAD by changing the doses, which establishes the required pathology and enables to study its impact on several cognitive areas. As the model is easy and affordable it can be used to study sporadic AD pathologies and develop its therapeutics [126].

#### APP knock-in rat

The major pathologies associated with AD are Aβ deposits, tau tangles, apoptotic death and neuroinflammation [5]. To mimic these pathologies in animals several transgenic mice models have been developed that exhibit AD pathologies to a great extent. But rarely these model show tau pathology, scarce neuronal death, and generate Aβ plaques in the brain areas not similar to that in human AD brains [127, 128]. Due to shortage of tools transgenic rat models have been of lesser interest in comparison to mice for modelling AD. Therefore, a CRISPR/Cas9-based APP knock-in rat line with Swedish-Beyreuther/Iberian-Arctic mutations has been developed. This rat model exhibits major AD pathologies that are uncommon in other transgenic mouse models. Inserting single chimeric APP gene has led to the development of reliable model that reflects pathologies associated with AD including Aβ build-up, astrocytosis and microgliosis resulting in neuroinflammation, tau hyperphosphorylation and apoptotic and necrotic neuronal death. These all ultimately proceed to synaptic decline and cognitive dysfunction [129]. Therefore, this model may aid in studying the pathogenesis of AD and may promote drug development.

## Conclusions

Animal models continue to play a crucial role in AD research. A most suitable model with pronounced AD pathology similar to that in human is required for development of novel therapeutics. Pathologically and biochemically animal models are quite comparable to the human disease conditions. The rodent models outperform the invertebrate ones in terms of neuroanatomy, the endocrine system and cognitive functions. Several chemicals, endogenous substances and heavy metals are there which are used to develop models that show similar AD pathologies that occur in humans. Chemicals like streptozotocin induce neuroinflammation along with other concomitant pathologies like oxidative stress, amyloid plaques deposition and tau hyperphosphorylation. Scopolamine induces cholinergic dysfunction which hinders signal transduction process resulting in memory loss. Colchicine and okadaic acid are involved majorly in hyperphosphorylation of tau protein. Endogenous substances like amyloid-β and acrolein promote amyloid-β aggregation and oxidative stress along with neuroinflammation respectively. Heavy metals generally lead to oxidative stress condition in animals along with neurofibrillary tangles formation. All these agents ultimately lead to the progression of AD and cognitive decline. Apart from chemical induced AD models, transgenic models have also been used to decipher disease pathology and develop novel treatment approaches. There are various knock-in and knock-out models targeting the crucial genes involved in AD progression. The major genes include APP, tau genes and PSEN genes. Transgenic models for overexpression of APP include PDAPP, APP23, Tg2576 and hAPP-J20. These models increase the production of Aβ aggregates along with other pathologies. P301S model is based on increasing the hyperphosphorylation of tau protein. There are some models which target multiple genes exhibiting multiple AD associated pathologies including APP/PS1, 3 × Tg and 5 × FAD. To advance the models used and to compensate the shortcomings of available models, novel AD models are under pipeline including oAβ25-35 model, transgenic model expressing 82-kDa ChAT, oDGal mouse and APP knock-in rat. These models can be further studied and used for various AD related evaluations regarding its pathology and developing therapy. Selection of animal model is very crucial for studying the disease pathology or evaluating the risk-benefits of a new compound before it can proceed to a clinical trial. Achieving a complete cure for diseases may not be realistic at the current stage. This is primarily due to the incomplete alignment between animal models and humans in terms of disease mechanisms. Therefore, the primary emphasis should be placed on the development of therapies geared towards disease modification, with the goal of slowing down disease progression. Additionally, proposing suitable animal models for this specific purpose would hold significant value. This study describes the most commonly used animal models and emerging reliable models of AD which may aid in developing novel potent drug candidates for the disease.

## Data Availability

Not Applicable.

## Abbreviations

3 × Tg: Triple transgenic mouse
5 × FAD: 5 AD linked mutation in Familial Alzheimer’s disease mouse
ADAM-10: A disintegrin and metalloproteinase domain containing protein-10
AGE: Advanced glycation end products
APP23: Amyloid Precursor Protein 23
ATG14: Autophagy related gene 14
BACE-1: β-Site APP cleaving enzyme
BChE: Butyrylcholinesterase
BDNF: Brain derived neurotrophic factor
Cas9: CRISPR associated protein-9
CAT: Catalase
ChAT: Choline acetyltransferase
COX-2: Cyclooxygenase-2
CRISPR: Clustered regularly interspaced short palindromic repeats
DMSO: Dimethyl sulfoxide
ERK1/2: Extracellular signal regulated kinase1/2
GPx1: Glutathione peroxidase-1
GSK-3β: Glycogen synthase kinase-3β
ICV: Intracerebroventricular
JNK: Janus kinase
LAMP-1: Lysosomal associated membrane protein-1
MAPK: Mitogen activated protein kinase
MAPT: Microtubule associated protein tau
MCI: Moderate cognitive impairment
MHC II: Major histocompatibility complex II
NFT: Neurofibrillary tangles
oDGal: Oligomeric D galactose
OKA: Okadaic acid
PDAPP: Human amyloid precursor protein V717F
PDGF: Platelet derived growth factor
PGC-1α: Peroxisome proliferator-activated receptor-gamma coactivator
PI3K: Phosphoinositide-3-kinase
SNARE: Soluble *N*-ethylmaleimide-sensitive factor activating protein receptor
SOD: Superoxide dismutase
STZ: Streptozotocin
TAA: Trans-arachidonic acid
TNF-α: Tumor necrosis factor-α

## References

[1] Götz J, Ittner LM (2008). Animal models of Alzheimer’s disease and frontotemporal dementia. Nat Rev Neurosci.

[2] Zhang XX, Tian Y, Wang ZT, Ma YH, Tan L, Yu JT (2021). The epidemiology of Alzheimer’s disease modifiable risk factors and prevention. J Prev Alzheimer’s Dis.

[3] Benedikz E, Kloskowska E, Winblad B (2009). The rat as an animal model of Alzheimer’s disease. J Cell Mol Med.

[4] Harikrishnareddy D, Misra S, Upadhyay S, Modi M, Medhi B (2015). Roots to start research in amyotrophic lateral sclerosis: molecular pathways and novel therapeutics for future. Rev Neurosci.

[5] Dhapola R, Hota SS, Sarma P, Bhattacharyya A, Medhi B, Reddy DH (2021). Recent advances in molecular pathways and therapeutic implications targeting neuroinflammation for Alzheimer’s disease. Inflammopharmacology.

[6] Thakur S, Dhapola R, Sarma P, Medhi B, Reddy DHK (2023). Neuroinflammation in Alzheimer’s disease: current progress in molecular signaling and therapeutics. Inflammation.

[7] Bhatti JS, Kaur S, Mishra J, Dibbanti H, Singh A, Reddy AP (2023). Targeting dynamin-related protein-1 as a potential therapeutic approach for mitochondrial dysfunction in Alzheimer’s disease. Biochim Biophys Acta Mol Basis Dis.

[8] Dhapola R, Sarma P, Medhi B, Prakash A, Reddy DH (2022). Recent advances in molecular pathways and therapeutic implications targeting mitochondrial dysfunction for Alzheimer’s disease. Mol Neurobiol.

[9] Nagar P, Sharma P, Dhapola R, Kumari S, Medhi B, HariKrishnaReddy D (2023). Endoplasmic reticulum stress in Alzheimer’s disease: molecular mechanisms and therapeutic prospects. Life Sci.

[10] Kumari S, Dhapola R, Reddy DHK (2023). Apoptosis in Alzheimer’s disease: insight into the signaling pathways and therapeutic avenues. Apoptosis.

[11] Beura SK, Dhapola R, Panigrahi AR, Yadav P, Reddy DH, Singh SK (2022). Redefining oxidative stress in Alzheimer’s disease: targeting platelet reactive oxygen species for novel therapeutic options. Life Sci.

[12] Beura SK, Dhapola R, Panigrahi AR, Yadav P, Kumar R, Reddy DH (2023). Antiplatelet drugs: potential therapeutic options for the management of neurodegenerative diseases. Med Res Rev.

[13] Janus C, Phinney AL, Chishti MA, Westaway D (2001). New developments in animal models of Alzheimer’s disease. Curr Neurol Neurosci Rep.

[14] Mullane K, Williams M (2019). Preclinical models of Alzheimer’s disease: relevance and translational validity. Curr Protoc Pharmacol.

[15] Grieb P (2016). Intracerebroventricular streptozotocin injections as a model of Alzheimer’s disease: in search of a relevant mechanism. Mol Neurobiol.

[16] Mostafavinia A, Amini A, Ghorishi SK, Pouriran R, Bayat M (2016). Laboratory animal research the effects of dosage and the routes of administrations of streptozotocin and alloxan on induction rate of typel diabetes mellitus and mortality rate in rats. Lab Anim Res.

[17] Kamat PK (2015). Streptozotocin induced Alzheimer’s disease like changes and the underlying neural degeneration and regeneration mechanism. Neural Regen Res.

[18] Ravelli KG, Rosário BD, Camarini R, Hernandes MS, Britto LR (2017). Intracerebroventricular streptozotocin as a model of Alzheimer’s disease: neurochemical and behavioral characterization in mice. Neurotox Res.

[19] Kadhim HJ, Al-Mumen H, Nahi HH, Hamidi SM (2022). Streptozotocin-induced Alzheimer’s disease investigation by one-dimensional plasmonic grating chip. Sci Rep.

[20] El-Shiekh RA, Ashour RM, Abd El-Haleim EA, Ahmed KA, Abdel-Sattar E (2020). *Hibiscus sabdariffa* L.: A potent natural neuroprotective agent for the prevention of streptozotocin-induced Alzheimer’s disease in mice. Biomed Pharmacother.

[21] Chen Y, Liang Z, Tian Z, Blanchard J, Dai CL, Chalbot S (2014). Intracerebroventricular streptozotocin exacerbates alzheimer-like changes of 3xTg-AD mice. Mol Neurobiol.

[22] Wei J, Yang F, Gong C, Shi X, Wang G (2019). Protective effect of daidzein against streptozotocin-induced Alzheimer’s disease via improving cognitive dysfunction and oxidative stress in rat model. J Biochem Mol Toxicol.

[23] Farbood Y, Sarkaki A, Mahdavinia M, Ghadiri A, Teimoori A, Seif F (2020). Protective effects of co-administration of zinc and selenium against streptozotocin-induced alzheimer’s disease: behavioral, mitochondrial oxidative stress, and GPR39 expression alterations in rats. Neurotox Res.

[24] Fronza MG, Baldinotti R, Martins MC, Goldani B, Dalberto BT, Schmitt Kremer F (2019). Rational design, cognition and neuropathology evaluation of QtC-4-MeOBnE in a streptozotocin-induced mouse model of sporadic Alzheimer’s disease. Sci Rep.

[25] Hajizadeh Moghaddam A, Ahmadnia H, Khanjani Jelodar S, Ranjbar M (2020). Hesperetin nanoparticles attenuate anxiogenic-like behavior and cerebral oxidative stress through the upregulation of antioxidant enzyme expression in experimental dementia of Alzheimer’s type. Neurol Res.

[26] Hira S, Saleem U, Anwar F, Sohail MF, Raza Z, Ahmad B (2019). β-carotene: a natural compound improves cognitive impairment and oxidative stress in a mouse model of streptozotocin-induced Alzheimer’s disease. Biomolecules.

[27] Saffari PM, Alijanpour S, Takzaree N, Sahebgharani M, Etemad-Moghadam S, Noorbakhsh F (2020). Metformin loaded phosphatidylserine nanoliposomes improve memory deficit and reduce neuroinflammation in streptozotocin-induced Alzheimer’s disease model. Life Sci.

[28] Akhtar A, Gupta SM, Dwivedi S, Kumar D, Shaikh MF, Negi A (2022). Preclinical models for Alzheimer’s disease: past, present, and future approaches. ACS Omega.

[29] Wang X, Chen M, Yang C, Liu X, Zhang L, Lan X (2011). Enhancing the scopolamine production in transgenic plants of Atropa belladonna by overexpressing pmt and h6h genes. Physiol Plant.

[30] Moharrami F, Hosseini B, Sharafi A, Farjaminezhad M (2017). Enhanced production of hyoscyamine and scopolamine from genetically transformed root culture of *Hyoscyamus reticulatus* L. elicited by iron oxide nanoparticles. In Vitro Cell Dev Biol Plant.

[31] Chavan RS, Supalkar KV, Sadar SS, Vyawahare NS (2023). Animal models of Alzheimer’s disease: an origin of innovative treatments and insight to the disease’s etiology. Brain Res.

[32] Chen WN, Yeong KY (2020). Scopolamine, a toxin-induced experimental model, used for research in Alzheimer’s disease. CNS Neurol Disord Drug Targets.

[33] Bajo R, Pusil S, López ME, Canuet L, Pereda E, Osipova D (2015). Scopolamine effects on functional brain connectivity: a pharmacological model of Alzheimer’s disease. Sci Rep.

[34] Tang KS (2019). The cellular and molecular processes associated with scopolamine-induced memory deficit: a model of Alzheimer’s biomarkers. Life Sci.

[35] Bhuvanendran S, Kumari Y, Othman I, Shaikh MF (2018). Amelioration of cognitive deficit by embelin in a scopolamine-induced Alzheimer’s disease-like condition in a rat model. Front Pharmacol.

[36] Rajashri K, Mudhol S, Serva Peddha M, Borse BB (2020). Neuroprotective effect of spice oleoresins on memory and cognitive impairment associated with scopolamine-induced Alzheimer’s disease in rats. ACS Omega.

[37] Anoush M, Pourmansouri Z, Javadi R, Ghorbanpour B, Sharafi A, Mohamadpour H (2022). Clavulanic acid: a novel potential agent in prevention and treatment of scopolamine-induced Alzheimer’s disease. ACS Omega.

[38] Nasiripour S, Zamani F, Farasatinasab M (2020). Can colchicine as an old anti-inflammatory agent be effective in COVID-19?. J Clin Pharmacol.

[39] Bakhta O, Blanchard S, Guihot AL, Tamareille S, Mirebeau-Prunier D, Jeannin P (2018). Cardioprotective role of colchicine against inflammatory injury in a rat model of acute myocardial infarction. J Cardiovasc Pharmacol Ther.

[40] Kumar A, Dogra S, Prakash A (2009). Neuroprotective effects of centella asiatica against intracerebroventricular colchicine-induced cognitive impairment and oxidative stress. Int J Alzheimers Dis.

[41] Sil S, Ghosh T (2016). Role of cox-2 mediated neuroinflammation on the neurodegeneration and cognitive impairments in colchicine induced rat model of Alzheimer’s disease. J Neuroimmunol.

[42] Karaduman Yesildal T, Karaduman T, Kutuk H. Alzheimer’s and huntington as neurodegenerative diseases. In: 4th International Symposium on Innovative Approaches in Engineering and Natural Sciences Proceedings 2019;4(6):101–103

[43] Saini N, Singh D, Sandhir R (2019). Bacopa monnieri prevents colchicine-induced dementia by anti-inflammatory action. Metab Brain Dis.

[44] Sil S, Ghosh T, Gupta P, Ghosh R, Kabir SN, Roy A (2016). Dual role of vitamin C on the neuroinflammation mediated neurodegeneration and memory impairments in colchicine induced rat model of Alzheimer disease. J Mol Neurosci.

[45] Essawy AE, Abdou HM, Ibrahim HM, Bouthahab NM (2019). Soybean isoflavone ameliorates cognitive impairment, neuroinflammation, and amyloid β accumulation in a rat model of Alzheimer’s disease. Environ Sci Pollut Res.

[46] Jiang X, Kumar M, Zhu Y (2018). Protective effect of hyperforin on β amyloid protein induced apoptosis in PC12 cells and colchicine induced Alzheimer’s disease: an anti-oxidant and anti-inflammatory therapy. J Oleo Sci.

[47] Kamat PK, Rai S, Swarnkar S, Shukla R, Nath C (2014). Molecular and cellular mechanism of okadaic acid (OKA)-induced neurotoxicity: a novel tool for Alzheimer’s disease therapeutic application. Mol Neurobiol.

[48] Koehler D, Williams FE (2018). Utilizing zebrafish and okadaic acid to study Alzheimer’s disease. Neural Regen Res.

[49] Yılmaz ŞG, Almasri S, Karabulut YY, Korkmaz M, Bucak Ö, Balcı SO (2023). Okadaic acid-induced Alzheimer’s in rat brain: phytochemical cucurbitacin E contributes to memory gain by reducing TAU protein accumulation. OMICS.

[50] Xu AH, Yang Y, Sun YX, Zhang CD (2018). Exogenous brain-derived neurotrophic factor attenuates cognitive impairment induced by okadaic acid in a rat model of Alzheimer’s disease. Neural Regen Res.

[51] Neha N, Sodhi RK, Jaggi AS, Singh N (2014). Animal models of dementia and cognitive dysfunction. Life Sci.

[52] Kamat PK, Nath C (2015). Okadaic acid: a tool to study regulatory mechanisms for neurodegeneration and regeneration in Alzheimer’s disease. Neural Regen Res.

[53] Kaushal A, Wani WY, Bal A, Gill KD, Kaur J (2019). Okadaic acid and hypoxia induced dementia model of Alzheimer’s type in rats. Neurotox Res.

[54] Budni J, de Oliveira J. Amyloid beta 1–42-induced animal model of dementia: a review. In: Colin R, Martin, Victor R, Preedy (editior) The Neuroscience of Dementia. Academic Press; 2020. p. 865–880.

[55] Zhang S, Wang P, Ren L, Hu C, Bi J (2016). Protective effect of melatonin on soluble Aβ1-42-induced memory impairment, astrogliosis, and synaptic dysfunction via the Musashi1/Notch1/Hes1 signaling pathway in the rat hippocampus. Alzheimer’s Res Ther.

[56] Shen WX, Chen JH, Lu JH, Peng YP, Qiu YH (2014). TGF-β1 protection against Aβ1–42-induced neuroinflammation and neurodegeneration in rats. Int J Mol Sci.

[57] Li Q, Che HX, Wang CC, Zhang LY, Ding L, Xue CH (2019). Cerebrosides from sea cucumber improved Aβ1-42 -induced cognitive deficiency in a rat model of Alzheimer’s disease. Mol Nutr Food Res.

[58] Samant NP, Gupta GL (2022). Avicularin attenuates memory impairment in rats with amyloid beta-induced Alzheimer’s disease. Neurotox Res.

[59] Kim HY, Lee DK, Chung B-R, Kim HV, Kim Y (2016). Intracerebroventricular injection of amyloid-β peptides in normal mice to acutely induce Alzheimer-like cognitive deficits. J Vis Exp.

[60] Swetha R, Kumar D, Gupta SK, Ganeshpurkar A, Singh R, Gutti G (2019). Multifunctional hybrid sulfonamides as novel therapeutic agents for Alzheimer’s disease. Future Med Chem.

[61] Rapaka D, Adiukwu PC, Bitra VR (2022). Experimentally induced animal models for cognitive dysfunction and Alzheimer’s disease. MethodsX.

[62] Chen C, Lu J, Peng W, Mak MS, Yang Y, Zhu Z (2022). Acrolein, an endogenous aldehyde induces Alzheimer’s disease-like pathologies in mice: a new sporadic AD animal model. Pharmacol Res.

[63] Zhang J, Sturla S, Lacroix C, Schwab C (2018). Gut microbial glycerol metabolism as an endogenous acrolein source. MBio.

[64] Huang YJ, Jin MH, Pi RB, Zhang JJ, Ouyang Y, Chao XJ (2013). Acrolein induces Alzheimer’s disease-like pathologies in vitro and in vivo. Toxicol Lett.

[65] Rashedinia M, Lari P, Abnous K, Hosseinzadeh H (2015). Protective effect of crocin on acrolein-induced tau phosphorylation in the rat brain. Acta Neurobiol Exp.

[66] Xu Q-Q, Yang W, Zhong M, Lin ZX, Gray NE, Xian YF (2023). Animal models of Alzheimer’s disease: preclinical insights and challenges. Acta Mater Medica.

[67] Bakulski KM, Seo YA, Hickman RC, Brandt D, Vadari HS, Hu H (2020). Heavy metals exposure and Alzheimer’s disease and related dementias. J Alzheimers Dis.

[68] Justin Thenmozhi A, Dhivyabharathi M, William Raja TR, Manivasagam T, Essa MM (2016). Tannoid principles of Emblica officinalis renovate cognitive deficits and attenuate amyloid pathologies against aluminum chloride induced rat model of Alzheimer’s disease. Nutr Neurosci.

[69] Cao Z, Yang X, Zhang H, Wang H, Huang W, Xu F (2016). Aluminum chloride induces neuroinflammation, loss of neuronal dendritic spine and cognition impairment in developing rat. Chemosphere.

[70] Mustafa HN (2020). Neuro-amelioration of cinnamaldehyde in aluminum-induced Alzheimer’s disease rat model. J Histotechnol.

[71] Thenmozhi AJ, Raja TRW, Janakiraman U, Manivasagam T (2015). Neuroprotective effect of hesperidin on aluminium chloride induced Alzheimer’s disease in wistar rats. Neurochem Res.

[72] Cao Z, Wang F, Xiu C, Zhang J, Li Y (2017). Hypericum perforatum extract attenuates behavioral, biochemical, and neurochemical abnormalities in aluminum chloride-induced Alzheimer’s disease rats. Biomed Pharmacother.

[73] Chiroma SM, Mohd Moklas MA, Mat Taib CN, Baharuldin MTH, Amon Z (2018). d-galactose and aluminium chloride induced rat model with cognitive impairments. Biomed Pharmacother.

[74] Zhang Y, Han X, Tang Y, Zhang J, Hu Z, Xu W (2022). Weakened interaction of ATG14 and the SNARE complex blocks autophagosome-lysosome fusion contributes to fluoride-induced developmental neurotoxicity. Ecotoxicol Environ Saf.

[75] Adedara IA, Abolaji AO, Idris UF, Olabiyi BF, Onibiyo EM, Ojuade TJD (2017). Neuroprotective influence of taurine on fluoride-induced biochemical and behavioral deficits in rats. Chem Biol Interact.

[76] Bartos M, Gumilar F, Bras C, Gallegos CE, Giannuzzi L, Cancela LM (2015). Neurobehavioural effects of exposure to fluoride in the earliest stages of rat development. Physiol Behav.

[77] Dec K, Łukomska A, Skonieczna-Żydecka K, Jakubczyk K, Tarnowski M, Lubkowska A (2020). Chronic exposure to fluoride affects GSH level and NOX4 expression in rat model of this element of neurotoxicity. Biomolecules.

[78] Hussien HM, Abd-Elmegied A, Ghareeb DA, Hafez HS, Ahmed HEA, El-moneam NA (2018). Neuroprotective effect of berberine against environmental heavy metals-induced neurotoxicity and Alzheimer’s-like disease in rats. Food Chem Toxicol.

[79] Raj K, Gupta GD, Singh S (2021). Spermine protects aluminium chloride and iron-induced neurotoxicity in rat model of Alzheimer’s disease via attenuation of tau phosphorylation, amyloid-β (1–42) and NF-κB pathway. Inflammopharmacology.

[80] Elder GA, Gama Sosa MA, De Gasperi R (2010). Transgenic mouse models of Alzheimer’s disease. Mt Sinai J Med.

[81] Schaeffer EL, Figueiró M, Gattaz WF (2011). Insights into Alzheimer disease pathogenesis from studies in transgenic animal models. Clinics.

[82] Zhao J, Fu Y, Yasvoina M, Shao P, Hitt B, O’Connor T (2007). β-Site amyloid precursor protein cleaving enzyme 1 levels become elevated in neurons around amyloid plaques: implications for Alzheimer’s disease pathogenesis. J Neurosci.

[83] Cai M. Research on the Pros and Cons of the Mouse Model of Alzheimer’s Disease. In: Proceedings of the 2022 International Conference on Creative Industry and Knowledge Economy (CIKE 2022). 2022.

[84] Van Dam D, Vloeberghs E, Abramowski D, Staufenbiel M, De Deyn PP. APP23 mice as a model of Alzheimer’s disease: an example of a transgenic approach to modeling a CNS disorder. CNS Spectr. 2005;10:207–22. 10.1017/s1092852900010051 15744222

[85] Sturchler-Pierrat C, Staufenbiel M (2000). Pathogenic mechanisms of Alzheimer’s disease analyzed in the APP23 transgenic mouse model. Ann N Y Acad Sci.

[86] Kawarabayashi T, Younkin LH, Saido TC, Shoji M, Ashe KH, Younkin SG (2001). Age-dependent changes in brain, CSF, and plasma amyloid β protein in the Tg2576 transgenic mouse model of Alzheimer’s disease. J Neurosci.

[87] Duan RS, Yang X, Chen ZG, Lu MO, Morris C, Winblad B (2008). Decreased fractalkine and increased IP-10 expression in aged brain of APPswe transgenic mice. Neurochem Res.

[88] Porcellotti S, Fanelli F, Fracassi A, Sepe S, Cecconi F, Bernardi C (2015). Oxidative stress during the progression of β-amyloid pathology in the neocortex of the Tg2576 mouse model of Alzheimer’s disease. Oxid Med Cell Longev.

[89] Bigl M, Apelt J, Eschrich K, Schliebs R (2003). Cortical glucose metabolism is altered in aged transgenic Tg2576 mice that demonstrate Alzheimer plaque pathology. J Neural Transm.

[90] Rogers K, Felsenstein KM, Hrdlicka L, Tu Z, Albayya F, Lee W (2012). Modulation of γ-secretase by EVP-0015962 reduces amyloid deposition and behavioral deficits in Tg2576 mice. Mol Neurodegener.

[91] Noda-Saita K, Terai K, Iwai A, Tsukamoto M, Shitaka Y, Kawabata S (2004). Exclusive association and simultaneous appearance of congophilic plaques and AT8-positive dystrophic neurites in Tg2576 mice suggest a mechanism of senile plaque formation and progression of neuritic dystrophy in Alzheimer’s disease. Acta Neuropathol.

[92] Boutajangout A, Goni F, Knudsen E, Schreiber F, Asuni A, Quartermain D (2009). Diminished Aβ burden in Tg2576 mice following a prophylactic oral immunization with a salmonella-based Aβ derivative vaccine. J Alzheimers Dis.

[93] Snellman A, Rokka J, Lopez-Picon FR, Eskola O, Wilson I, Farrar G (2012). Pharmacokinetics of [18F]flutemetamol in wild-type rodents and its binding to beta amyloid deposits in a mouse model of Alzheimer’s disease. Eur J Nucl Med Mol Imaging.

[94] Puzzo D, Gulisano W, Palmeri A, Arancio O (2015). Rodent models for Alzheimer’s disease drug discovery. Expert Opin Drug Discov.

[95] Chin J (2011). Selecting a mouse model of Alzheimer’s disease. Methods Mol Biol.

[96] Ameen-Ali KE, Simpson JE, Wharton SB, Heath PR, Sharp PS, Brezzo G (2019). The time course of recognition memory impairment and glial pathology in the hAPP-J20 mouse model of Alzheimer’s disease. J Alzheimers Dis.

[97] Shabir O, Sharp P, Rebollar MA, Boorman L, Howarth C, Wharton SB (2020). Enhanced cerebral blood volume under normobaric hyperoxia in the J20-hAPP mouse model of Alzheimer’s disease. Sci Rep.

[98] Hsu BY, Hung WL, Ho CT, Cheng IH, Hwang LS (2015). Protective effects of sesamol and ferulic acid on the formation of endogenous trans-arachidonic acid in hAPP J20 mice. J Funct Foods.

[99] Wright AL, Zinn R, Hohensinn B, Konen LM, Beynon SB, Tan RP (2013). Neuroinflammation and neuronal loss precede Aβ plaque deposition in the hAPP-J20 mouse model of Alzheimer’s disease. PLoS ONE.

[100] Allen B, Ingram E, Takao M, Smith MJ, Jakes R, Virdee K (2002). Abundant tau filaments and nonapoptotic neurodegeneration in transgenic mice expressing human P301S tau protein. J Neurosci.

[101] Yoshiyama Y, Higuchi M, Zhang B, Huang SM, Iwata N, Saido TCC (2007). Synapse loss and microglial activation precede tangles in a P301S tauopathy mouse model. Neuron.

[102] Savonenko A, Xu GM, Melnikova T, Morton JL, Gonzales V, Wong MPF (2005). Episodic-like memory deficits in the APPswe/PS1dE9 mouse model of Alzheimer’s disease: relationships to β-amyloid deposition and neurotransmitter abnormalities. Neurobiol Dis.

[103] Cai H, Wang Y, He J, Cai T, Wu J, Fang J (2017). Neuroprotective effects of bajijiasu against cognitive impairment induced by amyloid-β in APP/PS1 mice. Oncotarget.

[104] Locci A, Orellana H, Rodriguez G, Gottliebson M, McClarty B, Dominguez S (2021). Comparison of memory, affective behavior, and neuropathology in APPNLGF knock-in mice to 5xFAD and APP/PS1 mice. Behav Brain Res.

[105] Manook A, Yousefi BH, Willuweit A, Platzer S, Reder S, Voss A (2012). Small-animal PET imaging of amyloid-beta plaques with [11C]PiB and Its multi-modal validation in an APP/PS1 mouse model of Alzheimer’s disease. PLoS ONE.

[106] Li H, Wei Y, Wang Z, Wang Q (2015). Application of APP/PS1 transgenic mouse model for Alzheimer disease. J Alzheimers Dis Park.

[107] Javonillo DI, Tran KM, Phan J, Hingco E, Kramár EA, da Cunha C (2022). Systematic phenotyping and characterization of the 3xTg-AD mouse model of Alzheimer’s disease. Front Neurosci.

[108] Desai MK, Sudol KL, Janelsins MC, Mastrangelo MA, Frazer ME, Bowers WJ (2009). Triple-transgenic Alzheimer’s disease mice exhibit region-specific abnormalities in brain myelination patterns prior to appearance of amyloid and tau pathology. Glia.

[109] Shekari A, Fahnestock M (2021). Cholinergic neurodegeneration in Alzheimer disease mouse models. Handb Clin Neurol.

[110] Myers A, McGonigle P (2019). Overview of transgenic mouse models for Alzheimer’s disease. Curr Protoc Neurosci.

[111] Pietropaolo S, Feldon J, Yee BK (2014). Environmental enrichment eliminates the anxiety phenotypes in a triple transgenic mouse model of Alzheimer’s disease. Cogn Affect Behav Neurosci.

[112] Sasaguri H, Nilsson P, Hashimoto S, Nagata K, Saito T, De SB (2017). APP mouse models for Alzheimer’s disease preclinical studies. EMBO J.

[113] Oblak AL, Lin PB, Kotredes KP, Pandey RS, Garceau D, Williams HM (2021). Comprehensive evaluation of the 5XFAD mouse model for preclinical testing applications: a MODEL-AD study. Front Aging Neurosci.

[114] Oakley H, Cole SL, Logan S, Maus E, Shao P, Craft J (2006). Intraneuronal β-amyloid aggregates, neurodegeneration, and neuron loss in transgenic mice with five familial Alzheimer’s disease mutations: potential factors in amyloid plaque formation. J Neurosci.

[115] Eimer WA, Vassar R (2013). Neuron loss in the 5XFAD mouse model of Alzheimer’s disease correlates with intraneuronal Aβ42 accumulation and Caspase-3 activation. Mol Neurodegener.

[116] Kang S, Kim J, Chang KA (2021). Spatial memory deficiency early in 6xTg Alzheimer’s disease mouse model. Sci Reports.

[117] Pike CJ, Walencewicz-Wasserman AJ, Kosmoski J, Cribbs DH, Glabe CG, Cotman CW (1995). Structure-activity analyses of β-amyloid peptides: contributions of the β25–35 region to aggregation and neurotoxicity. J Neurochem.

[118] Yamada K, Nabeshima T (2000). Animal models of Alzheimer’s disease and evaluation of anti-dementia drugs. Pharmacol Ther.

[119] Canet G, Zussy C, Hernandez C, Maurice T, Desrumaux C, Givalois L (2023). The pathomimetic oAβ25–35 model of Alzheimer’s disease: potential for screening of new therapeutic agents. Pharmacol Ther.

[120] Chen ZR, Huang JB, Yang SL, Hong FF (2022). Role of cholinergic signaling in Alzheimer’s disease. Molecules.

[121] Gill SK, Ishak M, Dobransky T, Haroutunian V, Davis KL, Rylett RJ (2007). 82-kDa choline acetyltransferase is in nuclei of cholinergic neurons in human CNS and altered in aging and Alzheimer disease. Neurobiol Aging.

[122] Schliebs R, Arendt T (2011). The cholinergic system in aging and neuronal degeneration. Behav Brain Res.

[123] Braak H, Del Tredici K (2015). Neuroanatomy and pathology of sporadic Alzheimer’s disease. Adv Anat Embryol Cell Biol.

[124] Uribarri J, Cai W, Peppa M, Goodman S, Ferrucci L, Striker G (2007). Circulating glycotoxins and dietary advanced glycation endproducts: two links to inflammatory response, oxidative stress, and aging. J Gerontol A Biol Sci Med Sci.

[125] Xiao F, Li XG, Zhang XY, Hou JD, Lin LF, Gao Q (2011). Combined administration of D-galactose and aluminium induces Alzheimerlike lesions in brain. Neurosci Bull.

[126] Chadwick W, Maudsley S, Hull W, Havolli E, Boshoff E, Hill MDW (2023). The oDGal mouse: a novel, physiologically relevant rodent model of sporadic Alzheimer’s disease. Int J Mol Sci.

[127] Drummond E, Wisniewski T (2017). Alzheimer’s disease: experimental models and reality. Acta Neuropathol.

[128] Scearce-Levie K, Sanchez PE, Lewcock JW (2020). Leveraging preclinical models for the development of Alzheimer disease therapeutics. Nat Rev Drug Discov.

[129] Pang K, Jiang R, Zhang W, Yang Z, Li LL, Shimozawa M (2022). An App knock-in rat model for Alzheimer’s disease exhibiting Aβ and tau pathologies, neuronal death and cognitive impairments. Cell Res.

[130] Chesselet MF, Carmichael ST (2012). Animal models of neurological disorders. Neurotherapeutics.

[131] Moreira-Silva D, Carrettiero DC, Oliveira ASA, Rodrigues S, Dos S-L, Canas PM (2018). Anandamide effects in a streptozotocin-induced Alzheimer’s disease-like sporadic dementia in rats. Front Neurosci.

[132] Gilles C, Ertlé S (2000). Pharmacological models in Alzheimer’s disease research. Dialogues Clin Neurosci.

[133] Kumar A, Aggarwal A, Singh A, Naidu PS (2016). Animal models in drug discovery of Alzheimer’s disease: a mini review. EC Pharmacol Toxicol.

[134] Karaduman T, Kutuk H (2019). Alzheimer’s and Huntington as neurodegenerative diseases. Alzheimer’s Huntingt Neurodegener Dis.

[135] Yokoyama M, Kobayashi H, Tatsumi L, Tomita T (2022). Mouse models of Alzheimer’s disease. Front Mol Neurosci.

[136] Knezovic A, Osmanovic-Barilar J, Curlin M, Hof PR, Simic G, Riederer P (2015). Staging of cognitive deficits and neuropathological and ultrastructural changes in streptozotocin-induced rat model of Alzheimer’s disease. J Neural Transm.

[137] Santos TO, Mazucanti CHY, Xavier GF, Torrão AS (2012). Early and late neurodegeneration and memory disruption after intracerebroventricular streptozotocin. Physiol Behav.

[138] Joy T, Rao MS, Madhyastha S, Pai K. Effect of N-acetyl cysteine on intracerebroventricular colchicine induced cognitive deficits, beta amyloid pathology, and glial cells. Neurosci J. 2019:7547382. 10.1155/2019/7547382 PMC6500609 31139638

[139] Jadhav DD, Saraswat N. Evaluation of Neuroprotective effect of Cassia occidentalis L. against colchicine induced memory impairment in Wistar rats. Research square. 2023. 10.21203/rs.3.rs-3100202/v1.

[140] Çakır M, Yüksel F, Mustafa Özkut M, Durhan M, Kaymak E, Tekin S (2023). Neuroprotective effect of transient receptor potential Vanilloid 1 agonist capsaicin in Alzheimer’s disease model induced with okadaic acid. Int Immunopharmacol.

[141] Chou CH, Yang CR (2021). Neuroprotective studies of evodiamine in an okadaic acid-induced neurotoxicity. Int J Mol Sci.

[142] Li Y, Tian Q, Li Z, Dang M, Lin Y, Hou X (2019). Activation of Nrf2 signaling by sitagliptin and quercetin combination against β-amyloid induced Alzheimer’s disease in rats. Drug Dev Res.

[143] Zhang J, Ke KF, Liu Z, Qiu YH, Peng YP (2013). Th17 cell-mediated neuroinflammation is involved in neurodegeneration of Aβ1-42-induced Alzheimer’s disease model rats. PLoS ONE.

[144] Huang YJ, Zhang L, Shi LY, Wang YY, Yang YB, Ke B (2018). Caloric restriction ameliorates acrolein-induced neurotoxicity in rats. Neurotoxicology.

[145] Zhu Z, Lu J, Wang S, Peng W, Yang Y, Chen C (2022). Acrolein, an endogenous aldehyde induces synaptic dysfunction in vitro and in vivo: involvement of RhoA/ROCK2 pathway. Aging Cell.

[146] Chen X, Zhang M, Ahmed M, Surapaneni KM, Veeraraghavan VP, Arulselvan P (2021). Neuroprotective effects of ononin against the aluminium chloride-induced Alzheimer’s disease in rats. Saudi J Biol Sci.

[147] Adebiyi O, Adigun K, David-Odewumi P, Akindele U, Olayemi F (2022). Gallic and ascorbic acids supplementation alleviate cognitive deficits and neuropathological damage exerted by cadmium chloride in Wistar rats. Sci Rep.

[148] Nakai T, Yamada K, Mizoguchi H (2021). Alzheimer’s disease animal models: elucidation of biomarkers and therapeutic approaches for cognitive impairment. Int J Mol Sci.

[149] Bryan KJ, Lee HG, Perry G, Smith MA, Casadesus G. Transgenic mouse models of Alzheimer’s disease: behavioral testing and considerations. In: Buccafusco JJ, editor. Methods of Behavior Analysis in Neuroscience. 2nd ed. Boca Raton (FL): CRC Press/Taylor & Francis; 2009. 21204338

[150] Sturchler-Pierrat C, Abramowski D, Duke M, Wiederhold KH, Mistl C, Rothacher S (1997). Two amyloid precursor protein transgenic mouse models with Alzheimer disease-like pathology. Proc Natl Acad Sci USA.

[151] Kelly PH, Bondolfi L, Hunziker D, Schlecht HP, Carver K, Maguire E (2003). Progressive age-related impairment of cognitive behavior in APP23 transgenic mice. Neurobiol Aging.

[152] Oakley H, Cole SL, Logan S, Maus E, Shao P, Craft J (2006). Intraneuronal beta-amyloid aggregates, neurodegeneration, and neuron loss in transgenic mice with five familial Alzheimer’s disease mutations: potential factors in amyloid plaque formation. J Neurosci.

